# Prostate-Specific Antigen Reduction After Androgen Receptor Pathway Inhibitor Initiation: Real-World Comparison of Disease Progression Among Patients With Metastatic Castration-Sensitive Prostate Cancer

**DOI:** 10.36469/001c.141170

**Published:** 2025-07-29

**Authors:** Shawn Du, Carmine Rossi, Ibrahim Khilfeh, Porpong Boonmak, Gordon Wong, Dominic Pilon, Lorie Ellis

**Affiliations:** 1 Johnson & Johnson, Horsham, Pennsylvania; 2 Analysis Group, Inc., Montréal, Québec, Canada

**Keywords:** PSA reduction, castration resistance, overall survival, androgen receptor pathway inhibitors

## Abstract

**Background:** Prostate-specific antigen (PSA) has been used as both a screening tool and a marker for treatment response for advanced prostate cancer. With the introduction of androgen receptor pathway inhibitor (ARPI)-based treatment for metastatic castration-sensitive prostate cancer (mCSPC), there is a need to understand the impact that early treatment response, as measured by PSA, has on long-term clinical outcomes. **Objectives:** To assess whether long-term indicators of treatment success differ among ARPI-naïve patients with mCSPC who did or did not attain ≥90% reduction in PSA levels within 6 months of treatment initiation. **Methods:** Patients with mCSPC initiating a first ARPI (ie, apalutamide, enzalutamide, abiraterone acetate, darolutamide) were identified using electronic medical record data linked to insurance claims in the United States (1/1/2016–9/30/2022). Eligible patients were classified based on whether they achieved ≥90% reduction in PSA measured between pre-treatment and a window of 30 to 180 days after ARPI initiation. Cohorts were balanced using inverse probability of treatment weighting. Weighted Kaplan-Meier analysis was used to compare overall survival and castration-resistance–free survival by 36 months post-index between those with and without ≥90% PSA reduction. **Results:** Weighted cohorts included 1192 patients with early PSA reduction ≥90% and 699 without. By 36 months, significantly better overall survival was observed in those with early PSA reduction ≥90% than in those without (71.5% vs 54.7%; hazard ratio [HR]: 0.40, 95% confidence interval [CI]: 0.31, 0.50; *P*<.001). Similarly, significantly better castration-resistance–free survival was observed in those with early PSA reduction ≥90% than in those without (53.3% vs 36.8%; HR: 0.51, 95% CI: 0.43, 0.60; *P*< .001). **Discussion:** Early reduction of PSA levels by ≥90% within 6 months of ARPI initiation among patients with mCSPC in the real world is a robust indicator of treatment success, with improved long-term clinical outcomes, including survival and reduction in disease progression. **Conclusions:** These findings corroborate those of clinical trials and highlight the long-term benefits of an early and deep PSA response to ARPIs among real-world patients with mCSPC in the United States.

## BACKGROUND

Prostate cancer (PC) is the most common type of cancer among American men, with approximately 299 000 new cases estimated in 2024. Preventing tumor progression has always been the main goal of PC treatment, given that metastasis to vital organs is associated with a higher mortality rate. Prostate-specific antigen (PSA) decline is a valuable PC tumor marker that serves not only as a screening tool, but also as a treatment and disease progression monitoring tool. In particular, evaluation of PSA level before and soon after initiating treatment with androgen lowering therapies is one way of evaluating early therapeutic efficacy. In recent years, more effective androgen lowering therapies– androgen receptor pathway inhibitor (ARPI) regimens, including apalutamide, enzalutamide, abiraterone acetate plus prednisone, and darolutamide plus docetaxel, in combination with androgen deprivation therapy (ADT), have been approved for patients with metastatic castration-sensitive PC (mCSPC). In clinical trials of patients with mCSPC initiated on ADT, ARPIs have been associated with longer progression-free survival (PFS) and overall survival (OS), as well as improved PSA response, compared with ADT alone. Numerous studies ranging from phase III clinical trials to cooperative group and cohort trials have demonstrated a relationship between early, deep reduction of PSA and improved clinical outcomes, including long-term survival. However, there are currently limited data from large, real-world cohorts to confirm the association between early PSA reduction after ARPI initiation and long-term clinical outcomes, including OS. This study aimed to assess if long-term (ie, 36 months of follow-up) clinical outcomes, including OS, differ between patients who have an early, deep PSA response (ie, ≥90% PSA reduction) within 6 months of ARPI initiation compared with those who do not.

## METHODS

### Data Source

This study was conducted by linking common tokenized patient-level data within 2 data sources–a clinical database created from electronic medical records (EMR) and practice-record capture (PPS Analytics) and an administrative claims database (Komodo Research Database [KRD]) which included information between January 1, 2016, and September 30, 2022. The PPS Analytics database contains a wide range of demographic, clinical, and practice-record data, including PSA results, dispensation information, and medication and procedure data. The KRD is an insurance claims database of over 320 million patients in the United States (US) across commercial, Medicaid, and Medicare insurers, and includes information on insurance eligibility, diagnosis and procedures received in inpatient and outpatient settings, prescription fills, and mortality data, which are available from multiple third-party sources that aggregate their data from national and state governments, public listings, private claims, and obituary data. Data from PPS and KRD were linked by Datavant using their patent-pending, machine learning–validated, de-identification technology which replaces private patient information with an encrypted token that cannot be reverse-engineered to reveal the original information. The specific data sources used to capture ARPI treatment, castration resistance status, and metastasis in this study are provided in **Supplementary Table S1**. Data from both sources were de-identified and complied with the requirements of the Health Insurance Portability and Accountability Act of 1996, therefore, no review by an institutional review board was required.

### Study Design

A retrospective longitudinal design was used. Patients initiated on an ARPI (ie, apalutamide, abiraterone acetate, darolutamide, enzalutamide) were assigned into 2 mutually exclusive cohorts based on the change in PSA observed from pre- to post-ARPI initiation. The pre-ARPI PSA level was defined as the latest PSA measurement within 13 weeks before or at ARPI initiation. The post-ARPI PSA level was defined as the latest PSA measurement between 30 and 180 days (6 months) after ARPI initiation (ie, index date). Two cohorts were created based on the level of PSA reduction within 6 months of ARPI initiation: those with a deep, early PSA reduction ≥90% and those without. The baseline period was defined as the 12-month period preceding the index date. The observation period started on the index date and lasted until the latest of (1) the end of insurance eligibility, (2) the last date for a medical or pharmacy claim, or (3) the last recorded hospital discharge date, occurring no later than September 30, 2022 (end of the study period).

### Patient Selection Criteria

Adult males who were ARPI-naïve and initiated on their first ARPI on or after the date of approval for mCSPC by the US Food and Drug Administration were selected. Patients were required to have metastatic disease, in the absence of castration resistance, prior to the date of ARPI initiation. Metastatic disease was identified based on diagnosis codes for metastasis or clinical record in the EMR. Patients were also required to have ≥2 PSA results: one within 13 weeks before or on the date of ARPI initiation, and another between 30 and 180 days (6 months) following ARPI initiation. Patients were required to have ≥12 months of clinical activity preceding the index date. During the period between ARPI initiation and the index date, patients were also required to remain castration sensitive and be persistent on the index ARPI initiated, defined as having no gap in supply of ≥60 days. Patients were excluded if they had prior use of ARPI, immunotherapy, estrogens, radiopharmaceuticals, or poly (ADP-ribose) polymerase inhibitors before ARPI initiation. Concurrent use of ADT or corticosteroids with any ARPI was not required for inclusion in this study.

### Study Outcomes

During the observation period, clinical outcomes (ie, OS, time to castration resistance, castration-resistance–free survival [composite outcome including earliest of castration resistance or death]) were evaluated. Castration resistance was defined based on a diagnosis code for hormone-resistant malignancy, record of rising PSA after bilateral orchiectomy, or record of rising PSA after ≥90 days of continuous ADT use **(Supplementary Table S2)**. Patients were censored if they remained alive or did not have disease progression before the end of the observation period.

### Statistical Analysis

Baseline characteristics were described using means, standard deviations, and medians for continuous variables, and counts and proportions for categorical variables. To account for differences between the cohorts inverse probability of treatment weighting (IPTW) based on age, race, index ARPI, metastasis type, and baseline PSA level was applied to minimize confounding. Balance in baseline characteristics was evaluated using standardized differences, both before and after IPTW was implemented. Characteristics with standardized difference of <10% were considered balanced between cohorts. Comparisons between cohorts were assessed through weighted Kaplan-Meier (KM) graphs and rates, and weighted Cox proportional hazard models were used to calculate hazard ratios (HRs) with 95% confidence intervals (CIs) for clinical outcomes. In addition, we used a doubly-robust approach, where propensity score methods such as IPTW are used to minimize imbalances in measured covariates and, if there are still residual differences, covariates are adjusted in the outcome model. Specifically, all weighted models were further adjusted for time between metastasis and ARPI initiation and for time between ARPI initiation and the index date. The proportions of patients without disease progression through 36 months post index were also evaluated.

## RESULTS

### Patient Characteristics

**Figure 1.**
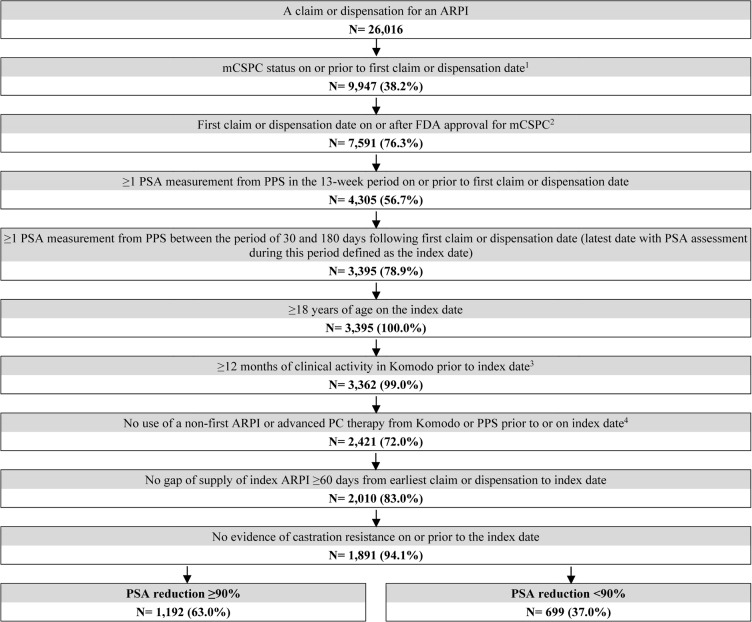
Sample Selection Flowchart Abbreviations: ARPI, androgen receptor pathway inhibitor; FDA, Food and Drug Administration; ICD-9/10-CM, International Classification of Diseases, Ninth/Tenth Revision, Clinical Modification; mCSPC, metastatic castration-sensitive prostate cancer; PC, prostate cancer; PSA, prostate-specific antigen. Notes: Metastatic disease was defined based on bone, nodal, or visceral metastasis ICD-9/10-CM diagnosis codes prior to or on the first claim or dispensation date. Patients were included in the if they had the indicator for metastasis, with the absence of an indicator for castration resistance, on or any time prior to the first claim or dispensation date. First claim or dispensation must have occurred on or after the approval date for mCSPC, which are as follows: Feb. 8, 2018, for abiraterone acetate, Sept. 17, 2019, for apalutamide, Dec. 16, 2019, for enzalutamide, and Aug. 5, 2022, for darolutamide.Insurance activity was determined based on the earliest and latest dates with insurance eligibility, medical claims, pharmacy claims, or inpatient-related claims in Komodo.Advanced PC-related medications included immunotherapy, estrogens, radiopharmaceuticals, and PARP inhibitors.

A total of 1192 patients with PSA reduction ≥90% and 699 patients without were selected **(Figure 1)**. Post weighting, demographic and baseline clinical characteristics were well balanced (ie, standardized difference <10%), except for time between metastasis and ARPI initiation, and time between ARPI initiation and index date **(Table 1)**. The mean age was approximately 73 years in both cohorts, with the majority being white patients. In both cohorts, abiraterone acetate was the most common ARPI patients initiated (38.4%), followed by apalutamide (32.7%) and enzalutamide (28.8%). The mean duration between ARPI initiation and the index date (ie, the date of the latest PSA evaluation by 6 months post-ARPI initiation) was 4.2 months for patients with PSA reduction ≥90% and 4.0 months for those without. The mean follow-up time was 16.9 months among patients with PSA reduction ≥90% and 14.3 months among those without.

**Table 1.** Baseline Characteristics

**Mean ± SD [Median] or n (%)**	**Unweighted**			**Weighted^1^**		
**PSA Reduction ≥90%** (**N = 1192)**	**PSA Reduction <90%** (**N = 699)**	**Standardized Difference (%)**	**PSA Reduction ≥90%^2^** (**N = 1192)**	**PSA Reduction <90%^2^** (**N = 699)**	**Standardized Difference (%)**	
Age, years	73.2 ± 8.6 [73.2]	73.7 ± 8.5 [73.7]	6.2	73.4 ± 8.5 [73.5]	73.8 ± 8.7 [73.8]	4.5
Race						
White	821 (68.9)	515 (73.7)	10.6	841 (70.6)	507 (72.5)	4.3
Black	220 (18.5)	96 (13.7)	12.9	198 (16.6)	103 (14.8)	5.0
Asian	15 (1.3)	5 (0.7)	5.5	15 (1.2)	4 (0.6)	6.6
Unknown	136 (11.4)	83 (11.9)	1.5	138 (11.6)	85 (12.1)	1.7
Payer						
Medicare	817 (68.5)	496 (71.0)	5.3	826 (69.3)	495 (70.8)	3.2
Commercial	287 (24.1)	149 (21.3)	6.6	279 (23.4)	148 (21.2)	5.3
Medicaid	34 (2.9)	16 (2.3)	3.6	32 (2.7)	18 (2.6)	0.8
Unknown	54 (4.5)	38 (5.4)	4.2	54 (4.5)	38 (5.4)	4.1
ARPI initiated						
Abiraterone acetate	428 (35.9)	294 (42.1)	12.6	452 (37.9)	274 (39.2)	2.6
Apalutamide	414 (34.7)	197 (28.2)	14.1	407 (34.2)	211 (30.1)	8.6
Enzalutamide	350 (29.4)	206 (29.5)	0.2	333 (27.9)	212 (30.4)	5.4
Darolutamide	0 (0.0)	2 (0.3)	7.6	0 (0.0)	2 (0.3)	7.6
Year of index date						
2018	70 (5.9)	43 (6.2)	1.2	76 (6.3)	42 (6.0)	1.6
2019	129 (10.8)	91 (13.0)	6.8	137 (11.5)	82 (11.7)	0.5
2020	274 (23.0)	149 (21.3)	4.0	271 (22.7)	150 (21.5)	3.1
2021	334 (28.0)	182 (26.0)	4.5	329 (27.6)	193 (27.6)	0.1
2022	385 (32.3)	234 (33.5)	2.5	380 (31.9)	233 (33.3)	3.1
Time between metastasis and ARPI initiation,^3^ mo	5.1 ± 13.4 [1.4]	11.1 ± 16.4 [4.5]	40.3	5.4 ± 14.1 [1.4]	10.2 ± 15.8 [4.0]	32.0
Time between ARPI initiation and index date,^3^ mo	4.2 ± 1.3 [4.3]	4.0 ± 1.4 [4.2]	12.0	4.2 ± 1.3 [4.3]	4.0 ± 1.4 [4.2]	13.9
De novo PC^4^	719 (60.3)	378 (54.1)	12.6	704 (59.1)	390 (55.7)	6.8
Metastasis type^5^						
Bone metastasis	834 (70.0)	525 (75.1)	11.5	857 (71.9)	511 (73.1)	2.8
Nodal metastasis	567 (47.6)	315 (45.1)	5.0	550 (46.2)	327 (46.8)	1.2
Visceral metastasis	199 (16.7)	141 (20.2)	9.0	213 (17.8)	133 (19.0)	3.0
Prior use of ADT^6^	1136 (95.3)	653 (93.4)	8.2	1136 (95.3)	651 (93.2)	9.4
Prior use of first-generation antiandrogens^6^	239 (20.1)	151 (21.6)	3.8	238 (20.0)	160 (22.9)	7.1
Prior use of bone antiresorptive therapy^7^	485 (40.7)	324 (46.4)	11.4	495 (41.5)	314 (45.0)	6.9
Prior chemotherapy^7^	19 (1.6)	22 (3.1)	10.2	20 (1.7)	20 (2.9)	7.9
Latest PSA level prior to ARPI initiation,^8^ ng/ml	49.3 ± 93.2 [10.0]	17.5 ± 57.2 [0.5]	41.1	38.0 ± 81.0 [7.2]	31.9 ± 79.2 [1.0]	7.5
Earliest Gleason score^9^						
≤6	67 (5.6)	33 (4.7)	4.1	69 (5.8)	31 (4.4)	6.5
7	210 (17.6)	116 (16.6)	2.7	213 (17.9)	109 (15.6)	6.1
8	180 (15.1)	110 (15.7)	1.8	180 (15.1)	104 (14.9)	0.7
9	274 (23.0)	143 (20.5)	6.1	273 (22.9)	145 (20.8)	5.2
10	45 (3.8)	43 (6.2)	11.0	47 (3.9)	41 (5.8)	9.0
Unknown	416 (34.9)	254 (36.3)	3.0	410 (34.4)	269 (38.5)	8.6

Abbreviations: ADT, androgen deprivation therapy; ARPI, androgen receptor pathway inhibitor; PC, prostate cancer; KM, Kaplan-Meier; PSA, prostate-specific antigen; SD, standard deviation.

Notes:

The weighted counts were rounded to the nearest integer and could result in total counts in a mutually exclusive category not adding up to 100%. Propensity scores were generated using probability estimates from a logistic regression model using the following predictors: age (continuous), Black race, type of ARPI initiated (abiraterone acetate vs. other), latest PSA level prior to ARPI initiation (as a continuous variable and categorized as ≥21 ng/ml vs. <21 ng/ml), bone metastasis, and visceral metastasis. Each patient was attributed an inverse probability of treatment weight that was defined as 1/(propensity score) for the patients with PSA reduction ≥90% and 1/(1-propensity score) for the patients with PSA reduction <90%. Normalized inverse probability of treatment weights were truncated at the 95th percentiles. Time between metastasis and ARPI initiation and time between ARPI initiation and index date were further used to adjust all KM analyses. Defined as the occurrence of metastasis no later than 180 after the initial PC diagnosis. Types of metastases were defined at any time prior to (and including) the date of first ARPI claim. Types of metastases were not mutually exclusive. Evaluated at any time prior to and excluding the index date. Evaluated during the 12-month baseline period (excluding the index date). Evaluated during the 12-month baseline period (including the index date). Evaluated any time prior to and including the index date.

### Clinical Outcomes

**Figure 2.**
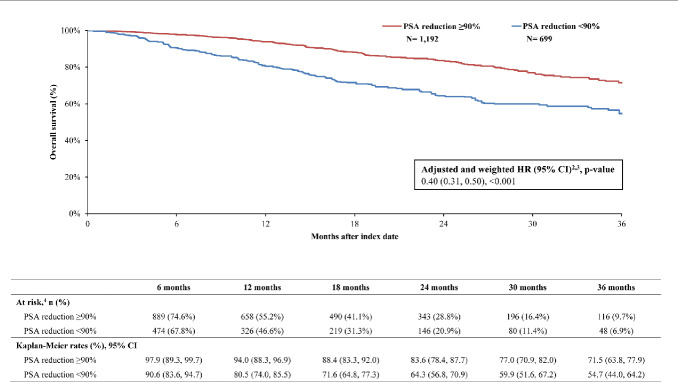
Overall Survival Among the Weighted Cohorts Abbreviations: ARPI, androgen receptor pathway inhibitor; CI, confidence interval; HR, hazard ratio; PSA, prostate-specific antigen. Notes: Overall survival was defined as the time from the index date to death (event) or the end of clinical activity (censored).Weighted HRs were based on Cox proportional hazard models comparing the cohort with PSA reduction ≥90% to the cohort with PSA reduction <90%. HR of <1 indicates a lower rate of death among the cohort with PSA reduction ≥90% compared with the cohort with PSA reduction <90%.Weighted HRs were additionally adjusted for the time between metastasis and ARPI initiation, and time between ARPI initiation and index date.This includes the number and percent of patients remaining at risk after the given period.

**Figure 3.**
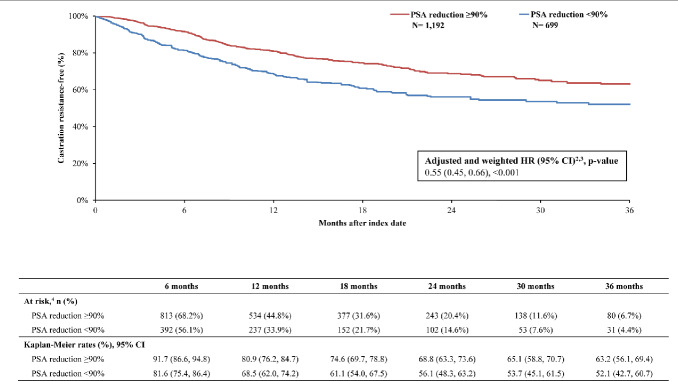
Progression to Castration Resistance Among the Weighted Cohorts Abbreviations: ARPI, androgen receptor pathway inhibitor; CI, confidence interval; HR, hazard ratio; PSA, prostate-specific antigen. Notes: Castration-resistance–free period was defined as the time from the index date to castration resistance (event) or the end of clinical activity (censored).Weighted HRs were based on Cox proportional hazard models comparing the cohort with PSA reduction ≥90% to the cohort with PSA reduction <90%. HR of <1 indicates a lower rate of castration resistance among the cohort with PSA reduction ≥90% compared with the cohort with PSA reduction <90%.Weighted HRs were additionally adjusted for the time between metastasis and ARPI initiation, and time between ARPI initiation and index date.This includes the number and percentage of patients remaining at risk after the given period.

**Figure 4.**
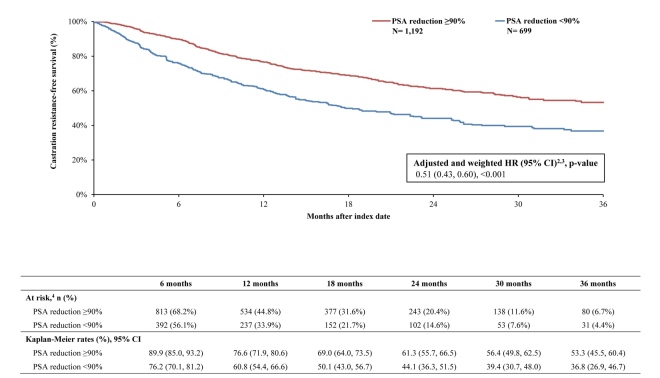
Castration-Resistance–Free Survival Among the Weighted Cohorts^1^ Abbreviations: ARPI, androgen receptor pathway inhibitor; CI, confidence interval; HR, hazard ratio; PSA, prostate-specific antigen. Notes: Castration-resistance–free survival was defined as the time from the index date to death or castration resistance (event) or the end of clinical activity (censored).Weighted HRs were based on Cox proportional hazard models comparing the cohort with PSA reduction ≥90% to the cohort with PSA reduction <90%. HR of <1 indicates a lower rate of castration resistance or death among the cohort with PSA reduction ≥90% compared with the cohort with PSA reduction <90%.Weighted HRs were additionally adjusted for the time between metastasis and ARPI initiation, and time between ARPI initiation and index date.This includes the number and percent of patients remaining at risk after the given period.

By 36 months, survival was greater in the PSA reduction ≥90% cohort (KM rate: 71.5%; 95% CI: 63.8, 77.9) compared with the cohort without PSA reduction ≥90% (KM rate: 54.7%; 95% CI: 44.0, 64.2; **Figure 2**). The likelihood of death was 60% lower overall in the cohort with PSA reduction ≥90% compared with the cohort without (HR: 0.40; 95% CI: 0.31, 0.50; *P*<.001). Additionally, differences in disease stabilization were observed; 63.2% (95% CI: 56.2, 69.4) of the cohort with PSA reduction ≥90% remained castration sensitive, while only 52.1% (95% CI: 42.7, 60.7) of the cohort without remained castration-sensitive (**Figure 3**). When PSA was reduced ≥90%, patients were 46% less likely to progress to castration resistance compared with those without this level of PSA reduction (HR: 0.55; 95% CI: 0.45, 0.66; *P*<.001). Taking both survival and castration resistance together, the castration-resistance–free survival rate by 36 months was greater among patients with PSA reduction ≥90% (KM rate: 53.3%; 95% CI: 45.5, 60.4; **Figure 4**) compared with patients without (KM rate: 36.8%; 95% CI: 26.9, 46.7). The likelihood of death or disease progression to castration resistance was 49% lower when PSA was reduced by ≥90% compared with those without the same level or reduction (HR: 0.51; 95% CI: 0.43, 0.60; *P*<.001).

## DISCUSSION

A deep, rapid PSA reduction within 6 months of initiating an ARPI was associated with longer survival time and delayed progression to castration resistance. These findings highlight the importance of early, deep PSA reduction as a critical early indicator of treatment effectiveness among patients with mCSPC.

The clinical benefits from achieving an early, deep PSA response observed in our study are consistent with those obtained in post hoc analyses of data from phase III clinical trials of ARPIs. In an analysis of data from the TITAN study, 78% of patients treated with apalutamide plus ADT had a deep PSA reduction (defined as ≥90% reduction or PSA ≤0.2 ng/ml) at the 3-month landmark time compared with only 33% of placebo plus ADT-treated patients. Among apalutamide-treated patients in the TITAN study, an early, deep PSA reduction at 3 months predicted longer OS (65% lower risk of death) and delayed time to castration resistance (62% lower risk of castration resistance) relative to no PSA reduction, which is aligned with the present study findings. Similarly, in an analysis of data from the LATITUDE study, a higher proportion of patients receiving abiraterone acetate and prednisone (AAP) plus ADT achieved PSA reduction ≥50% and ≥90% at any time relative to those receiving placebo plus ADT. Among patients treated with AAP plus ADT, the depth of PSA response at 6 months was associated with more favorable OS and radiographic PFS (rPFS). In a pattern consistent with the present study, AAP-treated patients with a PSA reduction ≥50% at ≤6 months post initiation had a 56% lower risk of death and a 74% lower risk of disease progression during the study period. In the SPARTAN study of patients with non-mCRPC, apalutamide also produced a deep and rapid PSA response along with associated long-term clinical benefits, suggesting a benefit among a broader population of patients with advanced PC disease.

In addition to the aforementioned clinical trials, our findings also align with other retrospective studies that documented a robust PSA decline after ARPI initiation and improved clinical outcomes among patients with mCSPC. In one study of 193 patients with mCSPC in Spain, apalutamide plus ADT induced a robust PSA response, with over 70% of patients achieving PSA reduction ≥90% at 3 months post initiation, as well as highly favorable OS and rPFS. Notably, apalutamide-treated patients with undetectable PSA levels (ie, ≤0.2 ng/ml) had a higher 18-month OS rates (98.7% and 65.3%, respectively) and rPFS rates (97.4% and 53.7%, respectively) than patients with detectable PSA (>0.2 ng/ml). Similarly, in a study of 108 patients with mCSPC in Japan, an initial per-label dose of apalutamide (ie, 240 mg/day) and a decline to undetectable PSA levels were significantly associated with delayed progression to castration-resistant disease. Although these prior real-world studies are informative, they were conducted using smaller sample sizes drawn from outside the US and relied solely on EMR data. Thus, the present findings add to this literature by documenting the long-term benefits of ARPI at 36 months of follow-up and the prognostic value of deep and rapid PSA responses in a larger, representative sample of patients with mCSPC in the US seleceted based on both EMR and insurance claims-based data.

Notably, other studies have suggested that a deep and early PSA response may be associated with favorable outcomes to subsequent lines of therapy. This implies that, even in cases where patients discontinue their initial ARPI regimen or transition to alternative treatments, an early, deep PSA response may serve as an independent indicator of prognosis and treatment success. Therefore, subsequent treatment modifications were not incorporated into the analytic framework. This approach was intended to isolate and characterize the association between achieving a PSA reduction ≥90% and subsequent clinical outcomes, regardless of later treatment changes.

Given the current findings and the existing body of evidence, an early, deep PSA response, particularly to ARPIs, is associated with improved survival and delayed progression among patients with mCSPC in both trial and real-world populations. Considering the demonstrated importance of PSA response and its association with clinical outcomes in the current study, the choice of the initial ARPI treatment may be an important consideration for treating physicians. For instance, prior studies among patients with mCSPC have demonstrated that apalutamide initiators experiencing earlier and deeper PSA reduction ≥90% relative to enzalutamide and abiraterone acetate initiators. Overall, the use of highly effective ARPI combined with prompt PSA monitoring may help physicians to gauge the response to treatment early and ensure the best possible clinical outcomes for patients with mCSPC.

### Limitations

This study may have been subject to certain limitations. For instance, EMR and claims databases may contain inaccuracies or omissions in PSA testing results, which were needed to assess inclusion criteria, treatment response, and study outcomes. Although comprehensive, linkages between PPS Analytics and KRD databases might have had mislinkages, potentially leading to misclassification and information bias. To control for this, we excluded patients that had inconsistent birth and death years between the 2 databases. An additional limitation was that certain patients may have received PSA testing outside of the urology network, in which case the results would not be captured. The present study used several well-established statistical methods to balance the cohorts with and without PSA reduction, including a doubly-robust approach that involves the use of propensity score methods (eg, IPTW) followed by multivariable regression to control for additional sources of confounding thereafter. However, as with any observational study, there remains a potential risk for confounding due to unobserved variables (eg, those that were unavailable in the claims or EMR data sources) or other biases (eg, miscoding or misclassification in the clinical record or through administrative claims may introduce selection and information biases) which could not be completely controlled for through weighting methods. Since all patients in the linked PPS Analytics were treated within community-based urology practices, results might not be generalizable to all patients with mCSPC in the US, including those in academic practices or practices other than urology (eg, oncology). Finally, this analysis relies on a single PSA reduction threshold (ie, PSA reduction ≥90%). While PSA reduction ≥90% has been established as a clinically meaningful marker of deep and early PSA response based on prior clinical trials of ARPI therapy, future real-world analyses can consider evaluating additional PSA thresholds.

## CONCLUSION

This real-world study assessed the association between PSA reduction and long-term clinical outcomes at 36 months of follow-up among patients with mCSPC initiated on ARPI in the US using combined EMR and claims databases. Early, and deep initial PSA response after ARPI initiation delayed progression to death and a more advanced disease stage among patients with mCSPC. These findings corroborate those of clinical trials and highlight the long-term benefits of an early and deep PSA response to ARPIs among real-world patients with mCSPC in the US.[Bibr ref-470622]

### Disclosures

S.D. and I.K. are employees and stockholders of Johnson & Johnson. C.R., P.B., G.W., and D.P. are employees of Analysis Group, Inc., a consulting company that has provided paid consulting services to Johnson & Johnson, which funded the development and conduct of this study.

### Ethics Statement

Data were de-identified and comply with the patient requirements of the Health Insurance Portability and Accountability Act (HIPAA) of 1996; therefore, no review by an institutional review board was required per 45 CFR §46.101(b)(4) (https://www.hhs.gov/ohrp/regulations-and-policy/regulations/45-cfr-46/#46.101).

## Data Availability

The data that support the findings of this study are available from PPS Analytics and Komodo Health Solutions. Restrictions apply to the availability of these data, which were used under license for this study.
